# Ectodomain Movements of an ATP-gated Ion Channel (P2X2 Receptor) Probed by Disulfide Locking*

**DOI:** 10.1074/jbc.M113.542811

**Published:** 2014-02-10

**Authors:** Olga Stelmashenko, Vincent Compan, Liam E. Browne, R. Alan North

**Affiliations:** From the ‡ Faculty of Medical and Human Sciences, and; the § Faculty of Life Sciences, University of Manchester, Oxford Road, Manchester M13 9PL, United Kingdom

**Keywords:** ATP, Disulfide, Ion Channels, Purinergic Receptor, Receptor Structure-function

## Abstract

The ectodomain of the P2X receptor is formed mainly from two- or three-stranded β-sheets provided symmetrically by each of the three subunits. These enclose a central cavity that is closed off furthest from the plasma membrane (the turret) and that joins with the transmembrane helices to form the ion permeation pathway. Comparison of closed and open crystal structures indicates that ATP binds in a pocket positioned between strands provided by different subunits and that this flexes the β-sheets of the lower body and enlarges the central cavity: this pulls apart the outer ends of the transmembrane helices and thereby opens an aperture, or gate, where they intersect within the membrane bilayer. In the present work, we examined this opening model by introducing pairs of cysteines into the rat P2X2 receptor that might form disulfide bonds within or between subunits. Receptors were expressed in human embryonic kidney cells, and disulfide formation was assessed by observing the effect of dithiothreitol on currents evoked by ATP. Substitutions in the turret (P90C, P89C/S97C), body wall (S65C/S190C, S65C/D315C) and the transmembrane domains (V48C/I328C, V51C/I328C, S54C/I328C) strongly inhibited ATP-evoked currents prior to reduction with dithiothreitol. Western blotting showed that these channels also formed predominately as dimers and/or trimers rather than monomers. The results strongly support the channel opening mechanism proposed on the basis of available crystal structures.

## Teaser

**Background:** The ATP-gated channel (P2X2) receptor ectodomain is formed by β-strands from three subunits.

**Results:** ATP-induced channel opening was prevented by disulfide locking between pairs of substituted cysteines in different subunits.

**Conclusion:** The turret region, the lower body wall, and the outer ends of transmembrane domains move significantly in channel opening.

**Significance:** Large ectodomain movements couple ATP binding to channel opening in P2X receptors.

## Introduction

P2X receptors assemble as trimers of the same (homomers) or different (heteromers) subunits. Each subunit has two membrane-spanning domains (TM1 and TM2)^2^ and a large ectodomain (1). ATP activates P2X receptors by binding to extracellular pockets formed between the ectodomains of pairs of the three subunits (2, 3). This drives a conformational change that leads to opening of a transmembrane ion permeation pathway. Crystal structures of closed (4) and open ATP-bound receptors (5) provide a compelling picture of how ATP binding might lead to pore opening. In the present work, we have sought to examine the opening mechanism by using functional recording of ion currents combined with disulfide engineering of P2X2 receptors.

The main body of the receptor ectodomain is formed from two- or three-stranded β-sheets, provided symmetrically by each of the three subunits (4). The lower body wall encloses a cavity, termed the central vestibule, which opens into the outer orifice of the transmembrane permeation pathway. The upper body wall, further from the plasma membrane, encloses a smaller but contiguous upper vestibule, and this closes into a central distal turret some 60 Å from the plasma membrane. Also projecting laterally from the upper body is the head domain, a disulfide-stabilized but otherwise rather poorly conserved structure of short strands and loops. Projecting laterally from the lower body wall are two “flippers” and the “dorsal fin”; these descriptors are derived from the overall dolphin-like appearance of a single P2X receptor subunit (4). The ATP-binding pocket is located ∼40 Å from the outer membrane surface: it is formed mostly by amino acid residues of the upper and lower body walls but also by the hydrophobic surface of the upper part of the dorsal fin. Comparing the closed (4) and open structures (5) indicates that ATP binding results in a retraction of the left flipper and an upward movement of the dorsal fin to approach the descending head domain. Together, these exert a lateral pull on the lower body, which expands markedly. This, in turn, leads to lateral displacement of the outer ends of the three transmembrane helices (particularly TM2 domains), and the resulting iris-like movement of these domains opens the ion-conducting pore.

P2X receptors have been studied extensively using cysteine substitution, most often in concert with tests for accessibility of thiol reactive groups of known size and charge (6–16). There have also been approaches based on disulfide engineering (3, 17–24). The aim of the present work was to introduce paired cysteine residues into positions that, from the closed and open structures, seemed appropriate to lock the receptor in a closed conformation that might be sensitive to oxidative release. We chose pairs in the turret region, the lower body wall, and the top of the TM domains.

## EXPERIMENTAL PROCEDURES

### 

#### 

##### Molecular and Cell Biology

Single and double cysteine mutations in rat P2X2 receptors were made as described previously (6, 9, 15, 17). Site-directed mutagenesis was performed on P2X2 receptor subunits using the Stratagene QuikChange method. The resulting mutation was PCR amplified with the appropriate 5′ and 3′ restriction sites on the PCR primer. All constructs were sequenced to confirm the coding region. Subunits were tagged with the EMYPME epitope at their C termini for use in Western blotting and biochemical detection of disulfide bonds. P2X2 subunits were transiently expressed together with GFP cDNA in HEK 293 cells by Lipofectamine 2000 (Invitrogen), using 25 ng/ml cDNA and 25 ng/ml GFP cDNA. Transfected cells were seeded on glass coverslips and used for experiments 24–48 h later.

##### Protein Biochemistry

48 h after transfection, cells were washed twice in phosphate-buffered saline containing 1 mm calcium, 0.5 mm magnesium, and 10 mm iodoacetamide. Proteins were solubilized in cell lysis buffer containing 20 mm Tris, pH 7.4; 100 mm NaCl; 5 mm EDTA; 1% nonylphenoxypolyethoxylethanol (Nonidet P-40); 10 mm iodoacetamide, and antiproteases and phosphatases (Halt Phosphatase Inhibitor Mixture; Pierce) for 30 min at 4 °C. After centrifugation (13,000 × *g*; 10 min), LDS sample buffer (Invitrogen) was added to the solubilized proteins, and the samples were boiled (95 °C for 5 min). Samples were separated on 4–12% NuPAGE gels (Invitrogen) and transferred to nitrocellulose membranes. Western blotting was performed according to standard protocols, and proteins were visualized using rabbit anti-EYMPME primary antibody (Bethyl Laboratories) and HRP-conjugated secondary antibody (both at 1:2000 dilution). Band densities were quantified using Carestream Molecular Imaging Molecular software.

##### Electrophysiological Recording

Whole-cell patch clamp recordings were made at room temperature 24–48 h after transfection at a holding potential of −60 mV. Patch pipettes were pulled from thin-walled borosilicate glass (World Precision Instruments) and had final resistances of 2–4 megohms. The basic extracellular solution contained the following (in mm): 147 NaCl, 2 KCl, 2 CaCl_2_, 1 MgCl_2_, 10 HEPES, and 13 d-glucose. ATP stock solutions were made in the extracellular solution, and the pH was adjusted to 7.3 using NaOH. The intracellular (pipette) solution contained the following: 145 mm NaCl, 10 mm HEPES, 10 mm EGTA. All solutions were maintained at pH 7.3 and 300–315 mOsm/liter. Chemicals were purchased from Sigma.

Currents were recorded with a patch clamp amplifier (Axopatch 200B) using pClamp 9 software (Molecular Devices). The data were low-pass filtered at 2 kHz and digitized at 5 kHz (whole-cell). Test compounds were applied with a RSC200 rapid perfusion system (Biologic, France). Between 60 and 80% of the series resistance was electronically compensated to minimize voltage errors in whole-cell recordings.

Two main protocols were used to investigate the effects of dithiothreitol (DTT). In the closed channel protocol, ATP was applied repeatedly (30 μm, 2 s) at 2-min intervals: after a control period, DTT (10 mm) was applied continuously for 10 to 20 min. In the open channel protocol, ATP was applied for 12 s, and DTT (10 mm) was co-applied for an 8-s period beginning 2 s after the start of the ATP application. Where stated, H_2_O_2_ was used at a concentration of 0.3%.

##### Data Analysis

Electrophysiological data were analyzed using Clampfit software (version 9; Molecular Devices) and Origin (version 8.2; OriginLab). Pooled data are presented as the mean ± S.E., and statistical significance was judged by Student's unpaired *t* test.

##### Homology Modeling

Homology models of the rat P2X2 receptor were made with MODELLER (25) (version 9.10) based on the closed and open zebrafish P2X4.1 crystal structures (Protein Data Bank codes 4DW0 and 4DW1). The lowest energy models were assessed with MolProbity (26) and minimized using AMBER (27). Models had 98.9% (closed) and 97.4% (open) residues in the allowed regions of the Ramachandran plot. Images were made in Chimera (version 1.62; 27).

## RESULTS

Cells expressing wild type P2X2 receptors responded to ATP (30 μm) with inward currents that declined by less than 20% during a 2-s application. There was no effect of DTT (10 mm, 20 min, *n* = 11). Most P2X2 receptors that contained a single cysteine substitution also responded to ATP (Table 1): those that did not were not studied further.

**TABLE 1:** **Summary of results for holding current, currents evoked by ATP (30 μm), and effects of dithiothreitol, with distance between atoms** * *p* < 0.05; ** *p* < 0.005. For double mutants, distances between the Cβ atoms in the same subunit are also shown in italics.

Receptor	*I*_hold_ (pA/pF)	*I*_ATP_ (pA/pF)	DTT (fold change)		Cβ-Cβ (Å)*^a^*	
			Closed	Open	Closed	Open
	*Mean* ± *S.E. (n)*	*Mean* ± *S.E. (n)*	*Mean* ± *S.E. (n)*	*Mean* ± *S.E. (n)*		
**Wild type**	1.7 ± 0.5 (11)	571 ± 105 (12)	0.83 ± 0.04 (11)	0.99 ± 0.04 (9)		
**Turret**						
P89C	5.6 ± 2.2 (4)	34.8 ± 10.9 (4)**	1.4 ± 0.2 (4)	1.1 ± 0.2 (4)	4.1	4.6
P89S	1.5 ± 0.3 (4)	155 ± 67 (4) **	0.85 ± 0.05 (4)	0.9 ± 0.09 (5)		
P90C	1.5 ± 0.3 (6)	40.7 ± 3.8 (4)**	8.5 ± 0.7 (4)**	1.1 ± 0.07 (5)	10.8	12.0
P90S	1.3 ± 0.3 (6)	621 ± 117 (6)	0.5 ± 0.07 (6)*	1.0 ± 0.04 (6)		
S97C	2.6 ± 1.1 (4)	315 ± 100 (4)	0.77 ± 0.4 (4)	1.0 ± 0.04 (4)	16.7	16.0
F291C	2.1 ± 0.3 (4)	26.5 ± 16.1 (4)**	1.9 ± 0.4 (4)	1.0 ± 0.04 (4)	13.5	13.6
P89C/S97C	1.0 ± 0.09 (5)	4.3 ± 1.0 (5)**	12.6 ± 2.1 (5)**	3.3 ± 0.7 (5)*	10.7	10.0
					*8.1*	*7.7*
P89C/F291C	1.5 ± 0.6 (5)	68.0 ± 32.5 (5)**	0.2 ± 0.02 (5)**	0.9 ± 0.06 (4)	8.7	8.4
					*8.6*	*9.5*

**Lower body wall**						
T60C	1.4 ± 0.4 (6)	246 ± 78 (6)*	2.2 ± 0.5 (6)*	2.5 ± 0.7 (6)*	7.6	14.1
T60S	1.9 ± 0.6 (4)	620 ± 150 (4)	0.88 ± 0.03 (4)	1.0 ± 0.08 (5)		
P62C	2.5 ± 0.7 (4)	313 ± 64 (4)*	0.59 ± 0.11 (4)	1.2 ± 0.2 (3)	14.4	20.3
S65C	1.7 ± 0.2 (4)	157 ± 108 (4)	0.66 ± 0.18 (4)*	1.0 ± 0.01 (4)	18.8	20.9
S190C	2.0 ± 0.7 (4)	259 ± 41 (4)*	0.8 ± 0.05 (4)	1.0 ± 0.02 (3)	26.1	28.7
H192C	1.3 ± 0.1 (5)	324 ± 79 (5)	0.66 ± 0.05 (5)*	0.9 ± 0.07 (3)	24.1	29.2
D315C	5.2 ± 3.2 (4)	197 ± 57 (4)**	0.39 ± 0.09 (4)*	1.0 ± 0.02 (4)	16.5	16.8
P62C/H192C	2.3 ± 0.4 (8)	29.5 ± 16.9 (8)**	6.0 ± 1.6 (8)*	2.4 ± 0.4 (5)*	16.8	22.1
					*7.3*	*7.6*
S65C/S190C	1.5 ± 0.1 (8)	34.4 ± 13.9 (8)**	3.5 ± 0.3 (8)**	2.9 ± 0.6 (7)*	21.7	24.2
					*5.0*	*4.8*
S65C/D315C	3.6 ± 0.9 (7)	122 ± 34 (7)**	3.2 ± 0.45 (7)**	2.8 ± 0.7 (4)	5.2	6.8
					*15.1*	*15.2*

**Outer end of TM**						
V48C	2.3 ± 0.9 (4)	600 ± 120 (4)	0.63 ± 0.05 (4)*	0.94 ± 0.01 (4)	18.6	21.8
V51C	2.4 ± 0.7 (4)	365 ± 97 (4)	0.81 ± 0.07 (4)*	1.0 ± 0.08 (4)	29.7	32.0
S54C	1.7 ± 1.0 (4)	619 ± 201 (4)	0.58 ± 0.2 (4)*	0.95 ± 0.03 (4)	14.9	21.6
I328C	4.2 ± 0.7 (5)*	742 ± 130 (5)	0.73 ± 0.05 (5)*	1.11 ± 0.05 (5)	15.6	27.9
V48C/I328C	16.1 ± 6.2 (5)	15.0 ± 3.1 (5)**	14.3 ± 3.5 (5)*	1.6 ± 0.2 (4)	5.4	14.6
					*14.5*	*14.9*
V51C/I328C	32.0 ± 8.6 (6)*	64.3 ± 19.1 (5)**	1.9 ± 0.3 (5)*	0.96 ± 0.02 (3)	10.3	15.2
					*20.0*	*20.0*
S54C/I328C	8.2 ± 2.2 (12)*	14.1 ± 5.4 (6)**	8.3 ± 2.4 (6)*	5.6 ± 0.9 (11)*	6.6	17.9
					*10.9*	*11.1*

*^a^* Mean of three measurements from each pair of subunits.

### Central Turret

#### 

##### Pro-89, Pro-90, Ser-97, and Phe-291

The part of the P2X receptor furthest from the plasma membrane forms a symmetrical turret from the loops between the β13 and β14 helices (at Gly-299 in the rat P2X2 receptor). The central aperture through this part of the protein is insufficiently large to admit ions (4, 5), which are considered to reach the extracellular vestibule through three lateral portals or fenestrations just above the plane of the membrane (5, 22, 28, 29).

**FIGURE 1.**
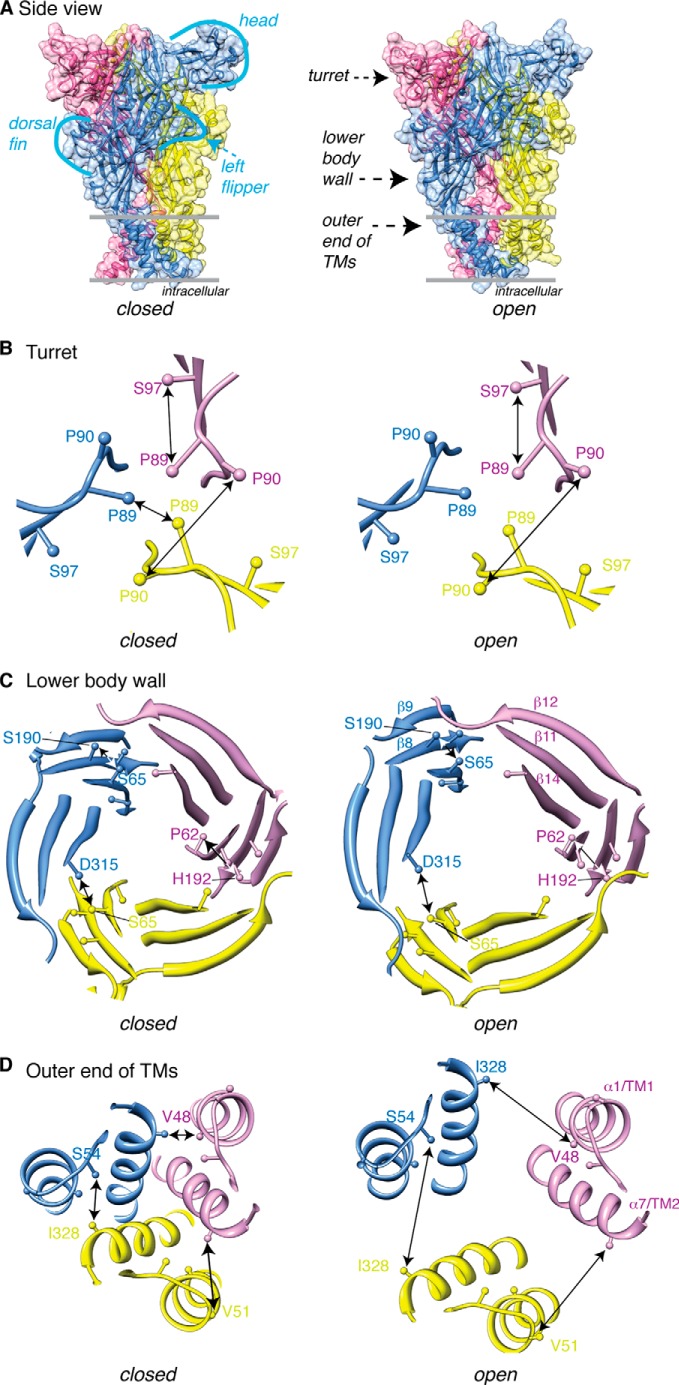
**P2X2 receptor homology models illustrate residues targeted for cysteine substitution.** The three subunits are shown in separate colors with *arrows* indicating the distances between residues in the closed (*left*) and open (*right*) receptor conformations. The *top panel* (*A*) shows a side profile of the P2X2 receptor in closed and open states, as it likely sits on the plasma membrane (*black lines*), and indicates the approximate positions of the residues studied. The central axis turret region (*B*), lower body wall (*C*), and outer end of the TMs (*D*) were studied.

A highly conserved proline residue (Pro-89 in P2X2 receptor) projects into and occludes the central permeation pathway in this central turret, separating the upper vestibule from the central vestibule (Pro-95 in zebra fish P2X4; Ref. 5). The Cβ atoms form a triangle with distances between them close to those observed in disulfide bonds (closed, 4.6 Å; open, 4.1 Å) (Fig. 1*A*). P2X2(P89C) receptors had normal holding currents, but currents in response to ATP (30 μm) were only about one-tenth that of wild type controls (or P2X2(P89S) (Table 1). This is consistent with a previous finding for the analogous P2X1 receptors (P93C; Ref. 14). DTT had no significant (<2-fold enhancement) or consistent effects on the ATP-evoked current.

**FIGURE 2.**
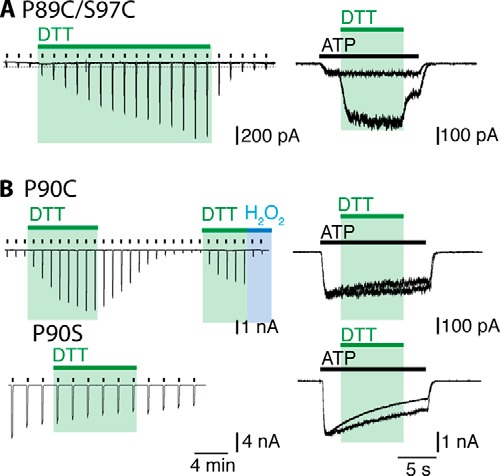
**Engineered disulfide bonds in the turret region limit P2X2 receptor function.** Recordings where made in the whole-cell configuration at a holding current of −60 mV and ATP (30 μm) was applied repeatedly every 2 min for 2 s (*left*), or for 12 s (*right*). DTT (10 mm) and H_2_O_2_ (0.3%) were applied where indicated. P2X2(P89C/S97C) receptors had small responses to ATP which were gradually increased by DTT application in the closed channel protocol and more rapidly in the open channel protocol (*A*). Currents from P2X2(P90C) receptors were enhanced by DTT only in the closed channel protocol (*B*). The current amplitude returned slowly to baseline, and this was more rapid in the presence of H_2_O_2_. Application of DTT to the (P90S) control had no effect on current amplitude. There was also clear enhancement with DTT in the open channel protocol (*C*, *right*). Representative recordings of *n* > 4 experiments are shown.

The side chain of Ser-97 projects into the lumen of this upper part of the central vestibule, at the start of the β4 strand that forms the lateral wall of the vestibule (4, 5). The distance (intersubunit) between the Cβ atoms is much too great to envisage disulfide formation (Table 1), and we found that P2X2(S97C) receptors had properties similar to wild type receptors. However, the double mutant P2X2(P89C/S97C) showed very small currents in response to ATP, but these small currents were greatly increased by DTT (10 mm) (Fig. 2*A*). The distance between the Cβ atoms of Pro-89 and Ser-97 in the same subunit (closed, 8.1 Å; open, 7.7 Å) is less than that between subunits (closed, 10.7 Å; open, 10.0 Å).

It has been reported previously that the P2X2(P89C/F291C) receptor is inhibited by DTT (20). We also found that DTT slowly reduced currents at this receptor (over a 10 min period). There was no effect when DTT was applied to the open channel during a 12-s application of ATP.

In contrast to Pro-89, the proline at position 90 in the P2X2 receptor is poorly conserved. P2X2(P90C) receptor showed normal holding currents, but ATP elicited very small currents (only about one-tenth those seen in the wild type channels, or P90S). DTT caused a 10-fold increase in the amplitude of the current evoked by ATP (30 μm) (Fig. 2*B*). This current amplitude returned to its low level during the 10 min following washout of DTT or much more rapidly when hydrogen peroxide was applied (0.3%) (Fig. 2*B*). DTT had no effect on the current when applied to the open channel during the ATP application (Fig. 2*B*): control studies showed that it also had no effect on ATP currents at P2X2(P90S) receptors (Fig. 2*B*). In neither the closed nor open structures do the Cβ atoms seem to be sufficiently close to form an intersubunit disulfide bond (closed, 10.8 Å; open, 12.0 Å).

### Lower Body Wall

#### 

##### Thr-60, Pro-62, Ser-65, Ser-190, His-192, and Asp-315

From the closed (4) and open (5) structures, it is considered that the binding of ATP leads to an upward movement of the dorsal fin so that it approaches the lower aspect of the head domain (5). This movement of the dorsal fin (which is formed mostly by the α3 helix) flexes the lower body domain outwards, and this motion is transmitted down to pull apart the outer ends of the TM helices. Each of the three subunits contributes two β-sheets to form the lower body wall: a two-stranded β sheet connecting to TM1 (β1 and β8: Thr-60, Pro-62, and Ser-65 are on β1; Ser-190 and His-192 are on β8) and a three-stranded β-sheet connecting to TM2 (β11, β12, and β14: Asp-315 is on β14) (Fig. 1*B*).

**FIGURE 3.**
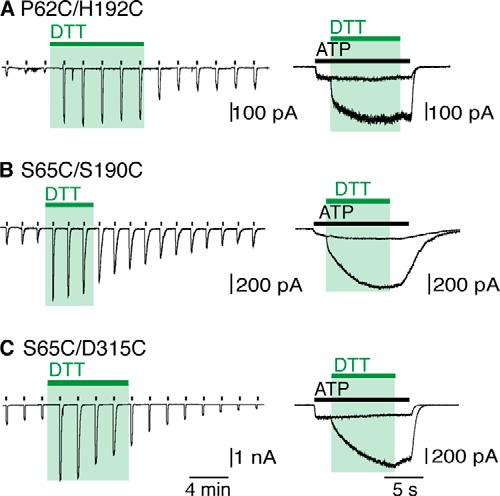
**Movement in the lower body wall in channel opening.** ATP (30 μm) was applied repeatedly every 2 min for 2 s (*left*), or for 12 s (*right*). DTT (10 mm) and H_2_O_2_ (0.3%) were applied where indicated. Responses to ATP were initially small for these receptors until DTT was applied. A 5-fold increase was seen for P2X2(P62C/H192C) in the closed channel protocol (*A*), and 3-fold increases in current amplitude was seen for (S65C/S190C) and (S65C/D315C) receptors (*B* and *C*, respectively). All three receptors displayed increased currents with DTT in the open channel protocol. Representative traces of *n* > 7 recordings are shown.

P2X2(P62C) and P2X2(H192C) receptors both gave currents similar to wild type channels, although of only ∼50% amplitude (313 ± 64 pA/pF (*n* = 4) and 324 ± 79 pA/pF (*n* = 4), respectively). However, the double mutant P2X2(P62C/H192C) gave very small currents in response to ATP (30 μm) (30 ± 17 (*n* = 8) pA/pF) (Fig. 3*A*), and these were greatly increased by DTT (10 mm) (Fig. 3*A*). The intrasubunit distance between the Cβ atoms of these two residues is 7.3 Å in the closed structure and 7.6 Å in the open channel: between chains the distances are 16.8 Å (closed) and 22.1 Å (open) (Table 1).

Likewise, Ser-65 and Ser-190 are adjacent to each other on two strands (β1 and β8, respectively) of the β-sheet that rises from the top of TM1. Their side chains are both oriented toward the inside of the lower body wall, and the distance (intrasubunit) between their Cβ atoms is close to that expected for disulfide bond formation when replaced by cysteine (closed, 5.0 Å; open, 4.8 Å). Replacement of both these residues by cysteine produced P2X2 receptors with very small currents, which were increased 5-fold by DTT (10 mm) (Fig. 3*B*). The effect of DTT occurred whether applied to the closed or open channel (Fig. 3*B*).

The side chain of Ser-65 also approaches that of Asp-315 on the β14 strand of an adjacent subunit. Indeed, a predicted intersubunit hydrogen bond involving the Oγ atom of Ser-65 and the Oδ2 atom of Asp-315 would break during channel opening (closed, 2.6 Å; open, 5.5 Å), thus allowing the lower body to expand (27). The substitution D315C did not change the holding current, but reduced the effect of ATP (30 μm) (Table 1). The double mutant receptor P2X2(S65C/D315C) had robust ATP-evoked currents, but these were increased 5-fold by DTT (10 mm) (Fig. 3*C*). This action of DTT was similar whether the channel was closed or open (Fig. 3*C*). The distances between the Cβ atoms are 5.2 Å (closed channel) and 6.8 Å (open channel).

The side chain of Thr-60 projects into central cavity at the lower part of the extracellular vestibule. The Cβ atoms are separated by 7.6 Å in the closed channel, and this increases to 14.1 Å when the channel opens. P2X2(T60C) receptor showed normal holding currents, and DTT (10 mm, 12 min) had a small effect (2-fold increase) on ATP-evoked currents (Table 1).

### Outer End of TMs

#### 

##### Val-48, Val-51, Ser-54, and Ile-328

**FIGURE 4.**
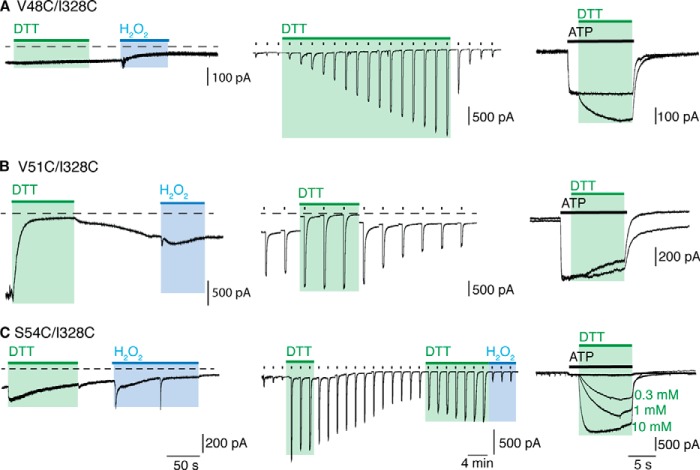
**P2X2 receptor opening and movement at the outer end of the TM domains.** DTT (10 mm) and H_2_O_2_ (0.3%) were applied in the absence of ATP (30 μm) (*left*). ATP was applied repeatedly every 2 min for 2 s (*center*), or for 12 s (*right*), with DTT and H_2_O_2_ applied where indicated. DTT had no effect on (V48C/I328C) receptors until ATP was present (*A*, *left*), whereby a gradual increase in current amplitude was observed (*A*, *center, right*). The large holding current of (V51C/I328C) receptors was rapidly reduced by DTT, and this was not fully recovered even in the presence of H_2_O_2_ (*B*, *left*). A reduced holding current with and increased ATP response was seen with DTT only in the closed channel protocol (*B*, *center*, *right*). A transient increase in holding current was seen with DTT for (S54C/I328C) receptors (*C*, *left*), and an increase in ATP-induced currents occurred in the closed channel protocol (*center*). A concentration-dependent increase in current amplitude with DTT was observed in the open channel protocol (*C*, *right*). Traces are representative of *n* > 5 recordings.

For Val-48 and Ile-328, the Cβ atoms are separated by 5.4 A and 14.6 Å in the closed and open channel structures (Fig. 1*C*) (4, 5). Each substitution alone provided receptors with large responses to ATP (30 μm) (in pA/pF: wild type, 571 ± 105 (*n* = 12); V48C, 600 ± 120 (*n* = 4); I328C, 742 ± 130 (*n* = 5). The holding current of V48C was not different from wild type (∼2 pA/pF), indicative of a low level of constitutive channel opening, and this was not changed by DTT. The holding current of I328C (4.2 ± 0.7 pA/pF, *n* = 5) was slightly increased as compared with wild type receptors (1.7 ± 0.5 pA/pF, *n* = 11). The double mutant (V48C/I328C) had a normal holding current (16 ± 6 pA/pF, *n* = 5), and this was unaltered by DTT (Fig. 4*A*, *left*). For P2X2(V48C/I328C) receptors, DTT (10 mm) elicited a large increase (14-fold) in the amplitude of current evoked by ATP (30 μm); this occurred progressively over tens of minutes (Fig. 4*A*, *center*). DTT also increased the current when applied to the open channel (in the presence of ATP): at 1 mm, the modification rate constant was 28 ± 8 m^−1^ s^−1^ (*n* = 4).

Val-51 lies almost one turn of the TM1 helix further out from Val-48. The most striking feature of the double mutation P2X2(V51C/I328C) was a large holding current in the absence of any applied ATP (32 ± 9 pA/pF, *n* = 6) (Fig. 4*B*, *left*). This was not observed with V51C or I328C alone (2.4 ± 0.7 (*n* = 4) and 4.2 ± 0.7 (*n* = 5) pA/pF, respectively). This large holding current was rapidly reversed by DTT (10 mm) and did not redevelop when the DTT was washed out, and neither when hydrogen peroxide was applied (Fig. 4, *left*). Continuous application of DTT also caused a small (∼2-fold) increase the responses to ATP, accompanied by a gradual reduction in holding current (Fig. 4, *center*). There was no effect of DTT (10 mm) when applied concomitantly with ATP. The distances between the Cβ atoms of Val-51 and Ile-328 are 10.3 Å and 15.2 Å, respectively, for the closed and open structure.

Ser-54 is situated on a loop at the very outer end of the TM1 helix, lying between two highly conserved residues (Lys-53 and Tyr-55). P2X2(S54C/I328C) receptors had holding currents slightly above normal, and these were not altered in any systematic way by DTT or H_2_O_2_ (Fig. 4*C*, *left*); the single mutant receptors S54C and I328C responded normally to ATP (Table 1). The double mutant S54C/I328C showed very small currents in response to ATP (30 μm) that were rapidly enhanced by application of DTT (Fig. 4*C*, *center*). The onset of action of DTT (10 mm) when applied to the open channel was 205 ± 21 m^−1^ s^−1^ (*n* = 11) (Fig. 4*C*, *right*). This implies that S54C is more accessible than V48C in the open channel, which is consistent with its position at the lower aspect of the lateral fenestration through which ions enter the permeation pathway (21, 27, 28).

##### Biochemical Evidence of Disulfide Bond Formation

**FIGURE 5.**
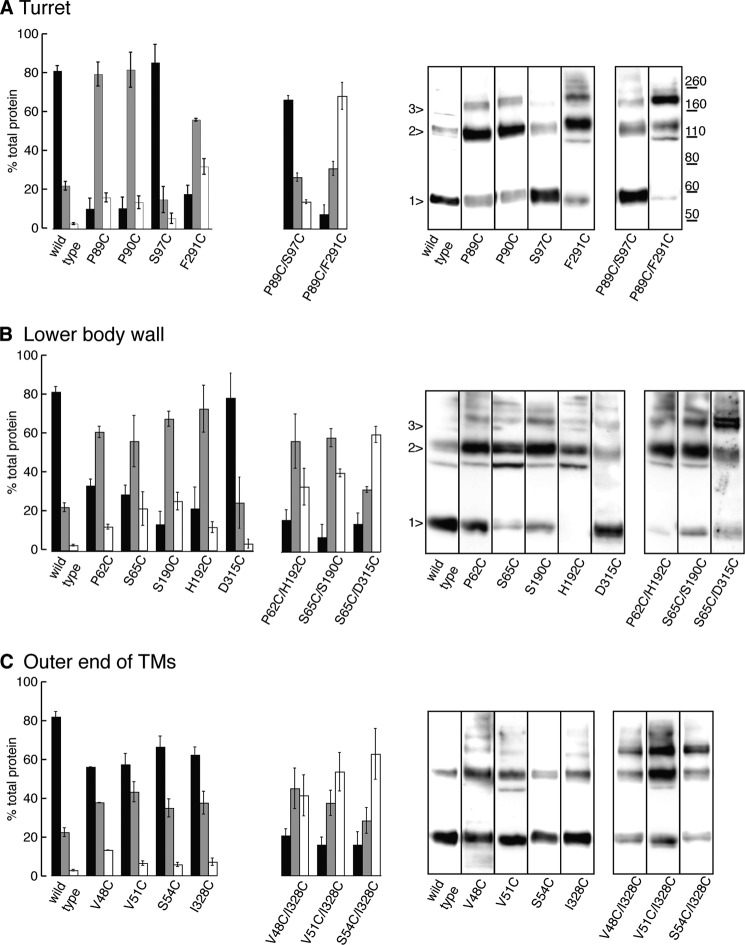
**Disulfide bond formation between P2X2 subunits after cysteine substitution.** The oligomeric state of P2X2 subunits was determined after cysteine substitution in the turret region (*A*), the lower body wall (*B*), and the outer end of the TM domains (*C*). Measurements of band intensity for monomers (*black bars*), dimers (*gray bars*) and trimers (*white bars*) of each mutant were made from non-reducing blots. Values represent percentage of the total protein and are means ± S.E. from three independent experiments. In each case, the *right* side shows representative Western blots from individual experiments.

Fig. 5 summarizes the biochemical experiments, with the oligomeric states for each mutant receptor represented as a percentage of the total protein. Wild type P2X2 receptors (same data shown in all three panels *A–C*) are predominantly expressed as monomers. In the turret region, introduction of a single cysteine at Pro-89 and Pro-90 resulted in a switch to higher order forms (dimers). This implies that disulfide bonds had formed between subunits, consistent with the observed effects of DTT in functional studies (Fig. 2*C*). The mutation S97C, either alone or together with P89C, did not alter the predominant form of the receptor protein. This suggests that the effect observed in functional studies with DTT (Fig. 2*A*) results from reduction of an intrasubunit disulfide bond. The distance between the Cβ atoms of Pro-89 and Ser-97 of the same subunit (∼8 Å) is significantly less than the distance between subunits (∼10.5 Å) (Table 1). In contrast, the double cysteine substitution P89C/F291C formed predominately trimers, but there was only a small inhibitory action of DTT: the Cβ atoms move little here between closed and open structures (8.7 Å and 8.4 Å, respectively).

All of the three double cysteine substitutions (P62C/H192C, S65C/S190C, S65C/D315C) existed in predominately dimeric and trimeric form, although in the first two cases, the Cβ distances in the fully folded channel are too great for disulfide formation (Table 1). The double mutant S65C/D315C was mostly dimeric and trimeric, although each of the single mutant receptors was mostly monomeric. The Cβ distance between Ser-65 and Asp-315 for the closed channel is consistent with that observed in disulfide bonds and increases during channel opening (Table 1). P2X2 receptors carrying single cysteines near the outer end of the TM domains remained predominately monomeric, but introduction of two cysteines in each case resulted in the receptor appearing mostly as dimer or trimer (Fig. 5*C*).

## DISCUSSION

An aim of this study was to determine whether the closed and open structures determined for purified protein by crystallography were consistent with the behavior of the protein in a cell membrane. The approach is made possible in P2X receptors by the absence of any effect of reducing agents on the function of the wild type P2X2 receptor when expressed in HEK cells, despite the existence of five conserved disulfides in the ectodomain (6, 7). The interpretation of the results must also take into consideration other factors. First, calculations of the distance between the Cβ atoms in a disulfide bond (30) must be compared with the distances computed from relatively stable closed and open states. In the transition from one to the other, the channel may pass through intermediate states in which these distances differ. Likewise, although the closed and open structures exhibit 3-fold symmetry, the cysteine-substituted channel may adopt “asymmetrical” conformations due to one pair that is disulfide locked, but still operate functionally. Second, it should be borne in mind that the resolution of the structures on which our models are based was 2.9 and 3.0 Å, which allows potentially for considerable side chain flexibility. Third, one must consider that replacement of a naturally occurring amino acid with cysteine might itself lead to more general change in folding and structure that would invalidate simplistic interpretations based on distances measured between Cβ atoms.

This third limitation merits further discussion. Several of the cysteine-substituted receptors had currents in response to ATP (30 μm) that were much smaller than those observed in wild type channels (Table 1). There are two obvious explanations. First, that the correct folding and trafficking of the channels to the plasma membrane has been impaired by the mutation(s): such an explanation invalidates the approach and no further conclusions can be drawn. Second, that the folding and trafficking are essentially normal, and the small current results from an ectopic disulfide bond that prevents channel opening. The evidence for this explanation came from the large effect of DTT, where a very small initial ATP-induced current was increased as much as 15-fold by 1 mm DTT. The effects of DTT differed greatly in their time course of onset, being rapid in some cases (S54C/I328C (Fig. 4), P62C/H192C, S65C/S190C, S65C/D315C (Fig. 3)), and slow in others (P89C/S97C (Fig. 1) and V48C/I328C (Fig. 4). It was not feasible to explore complete ATP concentration-response curves in the context of such changing baseline currents. Nevertheless, for each major region studied (turret, lower body wall, outer end of TM) examples were found which allow the conclusion that disulfide bond breakage was required for “normal” channel opening.

From the closed and open structures, the central turret of the receptor forms a scaffold against which the sides of the lower body wall can flex outwards during channel opening (5). In this central turret, Pro-89 is highly conserved among P2X receptors. The Cβ atoms of the Pro-89 residues provided by different subunits indicate that cysteines in this position would be sufficiently close to form disulfide bonds (Table 1), particularly when taking into account the increased flexibility of side chain orientation that would accompany the replacement of proline by cysteine. Consistent with this, the majority of these channels formed as dimers rather than monomers (Fig. 5*A*). Although ATP-induced currents were reduced in amplitude in the P2X2(P89C) channel, the results suggest that the proline itself is not involved in any hinge-like movement, but rather contributes to the formation of a compact scaffold. It also suggests that an asymmetrical receptor with one disulfide and one free cysteine at this position can function, if not optimally. For the P89C/S97C double mutant, the ATP currents were increased in both the closed channel and open channel protocol (Fig. 2*A*). The finding that the P2X2(P89C/S97C) receptor remained predominately monomeric suggests that this disulfide bond is intrasubunit. The calculated Cβ distances for these residues are less within the same subunit than between different subunits, but they are nonetheless greater than are consistent with disulfide formation (Table 1). This may suggest that the closed to open transition passes through an intermediate conformation in which these residues become closer.

In the case of Pro-90, which is not well conserved, DTT increased ATP-induced currents when applied to the closed channel. The effect was relatively slow, but was clearly due to disulfide reduction: it was rapidly reversed by oxidation with H_2_O_2_, and it was not observed with P2X2(P90S) receptors. No effect was observed when DTT was applied to the open channel. This suggests that closed channel conformational movements in the P90C receptor bring these side chains sufficiently close to undergo disulfide formation.

In the region of the lower body wall (Fig. 1*B*), we sought evidence that movements occurred between the component β-strands as the wall flexes outwards. The side chains of Pro-62 and Ser-65 are oriented on opposite sides of the β1 strand that rises from the outer end of the TM1 and forms the base of the ATP binding pocket (residues Lys-69 and Lys-71). In the case of Pro-62 and His-192 individually, the currents evoked by ATP were in each case substantial (Table 1). However, they were much less for the double mutant P62C/H192C until the application of DTT (Fig. 3*A*). The large (6-fold) increase in current produced by DTT strongly suggests that the disulfide bridge at this position (which must be intrasubunit, Fig. 1*B* and Table 1) significantly impairs the closed-open transition. A similar interpretation pertains to the S65C/S190C pair. This finding is surprising, because no substantial intrasubunit rearrangement is expected between β-strands on account of their rigid stabilization by hydrogen bonds. Whereas Pro-62 lies on the β1 strand that forms the base of the ATP binding pocket (at Lys-69 and Lys-71), His-192 is situated at the end of the β8 strand just following the highly conserved N^182^FTILIKN: neither residues are well conserved among P2X receptors. Taken together with the intrasubunit distance between the Cβ atoms of P62C/H192C (closed, 7.3 Å; open, 7.6 Å: Table 1) and S65C/S190C (closed, 5.0 Å; open, 4.8 Å: Table 1) these effects of DTT suggest that locking the side chains of these residues together is sufficient to impair the movement of the body wall β-strand during channel opening.

No such reasoning is required in the case of S65C/D315C pair. The Cβ distances (closed, 5.2 Å; open, 6.8 Å: Table 1) in this case are in the range that might be expected for an intersubunit disulfide bond to prevent channel opening. D315C is situated on the long β14 strand that runs from the turret of the receptor to near the end of second transmembrane domain, and which contributes to the ATP binding pocket (Lys-308, Arg-313) (4).

Our results from the region at the outer end of the second transmembrane confirm and expand those observed previously for the V48C/I328C pair (17). In the case of V48C and S54C, the closed Cβ distances are in the range for disulfide bond formation in the closed but not the open state. In both these cases, application of DTT caused a large increase in the ATP-evoked currents, and the more rapid effect in the case of S54C is consistent with the relatively more exposed situation of this residue (4, 5, 31). The case of V51C/I328C is more difficult to interpret given the very large holding currents, indicative of constitutive opening, and the relatively smaller effect of DTT (Table 1) that we observed with this substitution. In view of the predicted distance between these residues (Cβ atoms, 10.3 (closed) and 15.2 (open)), this result suggests that (at least a proportion of) channels are assembled into a state distinct from that expected from the crystal structures.

The main conclusion of our work is that the principal movements associated with P2X channel opening are a reorientation of the subunits with respect to each other, rather than major structural rearrangement within individual subunits. This is consistent with previous work on P2X receptors using a similar approach (17–24, reviewed in Ref. 32), and reflects the situation for pentameric (33) and tetrameric (34) ligand-gated channels. There are reasons to question whether the two available crystal structures provide an accurate account of how the protein operates in the plasma membrane. For example, highly conserved cytoplasmic domains have been removed in the preparation of crystal formation (4, 5), and molecular dynamics simulations suggest that the large intersubunit crevices are not present in the membrane-embedded receptor (35). Nonetheless, the present results with disulfide locking, for the turret, the lower body wall, and the outer end of the transmembrane domains, are generally consistent with the closed to open movements predicted from the crystal structures.

## Footnotes

2 The abbreviation used is:

TM: transmembrane domain.
